# Effects of Personal Ability and Social Welfare on the Health of Rural-to-Urban Elderly Migrants: An Empirical Analysis from Jiangsu Province, China

**DOI:** 10.3390/healthcare9121760

**Published:** 2021-12-20

**Authors:** Xinglong Xu, Yupeng Cui, Yang Cai, Henry Asante Antwi, Lulin Zhou, Joseph Noah Bangura

**Affiliations:** 1 School of Management, Jiangsu University, Zhenjiang 212013, China; 2211910057@stmail.ujs.edu.cn (Y.C.); 2211910056@stmail.ujs.edu.cn (Y.C.); 5103150217@stmail.ujs.edu.cn (H.A.A.); 1000004207@ujs.edu.cn (L.Z.); 2 Zhenjiang Association of Gerontology and Geriatrics, Zhenjiang 212013, China

**Keywords:** rural-to-urban elderly migrants, health vulnerability, personal ability, social welfare

## Abstract

This study aims to discuss the relationship between personal endowment and social welfare on the health status of the rural-to-urban elderly migrants. It constructed the theoretical framework of the health vulnerability of rural-to-urban elderly migrants. The health status of rural-to-urban elderly migrants was divided into three dimensions: physical health, mental health, and social adaptation. A total of 658 rural-to-urban elderly migrants in 12 cities of Jiangsu province were selected as samples for empirical test and analyzed the influence of individual endowments and social welfare on the health status of rural-to-urban elderly migrants and their differences. The result shows that personal ability affects the social adaptation ability of rural-to-urban elderly migrants, and social welfare has a significant influence on the physical and mental health of rural-to-urban elderly migrants. Lacking the learning ability of rural-to-urban elderly migrants in sample areas is the main factor that leads to their low social adaptation ability and the unequal social welfare and public services restricting the physiological and mental health status of rural-to-urban elderly migrants.

## 1. Introduction

Population aging has become a major social problem across the world today, especially in advanced and emerging economies., which leads to a series of issues, such as the increasing prevalence of chronic diseases, difficulty in self-care, high disability rate, and high mortality rate among the elderly, especially in rural areas of China [1]. For a long time, China’s urban–rural dual structure has widened the inequality of urban–rural common service resources. Due to the poor accessibility of medical and health service resources, coupled with the obvious empty nest phenomenon in rural areas, many older adults lack sufficient health knowledge, which has seriously affected the health status of the elderly. Therefore, with economic and social development, more and more rural elderly move to cities with their children [2]. According to the 2018 China’s floating population development report, the scale of China’s elderly floating population increased from 5.03 million in 2000 to 13.04 million in 2015, with an average annual growth of 6.6%. When the rural elderly move to cities, they obtain more medical and health resources. However, the social welfare policies and public service systems of rural-to-urban elderly migrants. such as economic support, medical insurance, spiritual comfort, etc., are not yet well developed and have been in a long-term absence or low-level operation [3,4,5]. Rural-to-urban elderly migrants cannot become real citizens, and their socal welfare is inconsistent with the social welfare obtained by urban residents.

Existing studies have shown that the inconsistent personal endowments and unequal social welfare policies for the rural-to-urban elderly migrants after moving from the countryside to the city are important factors that differentiate their physical or psychological problems [6]. However, few scholars have combined the two to analyze the health status of the rural-to-urban elderly migrants, and the few related ones are without a systematic and effective theoretical analysis framework. As a result, the particulars, circumstances, characteristics, attributes, and the specific fact of details of the health status of the elderly in rural areas remain unclear. Further, deconstruction of the extant literature reveals that previous studies on the impact of the health status of the rural-to-urban elderly migrants still have the following aspects to be deepened. Firstly, most of the research on the health status of the rural-to-urban elderly migrants focuses on the changes in human capital and the accessibility of medical resources [7,8]. Many scholars also recognize the important intermediary role of personal endowments. However, few scholars have conducted an in-depth analysis on how personal endowment affects the health of rural-to-urban elderly migrants. Secondly, a substantial number of the existing research on the impact of social welfare on the health status of the rural-to-urban elderly migrants focuses on qualitative research, thus limiting the extent of details revealed [9]. Since the social welfare policy of the migrant population is a gradual improvement process, its impact on the health of the rural-to-urban elderly migrants needs to be evaluated with the latest data to ensure the objectivity and dynamics of the research conclusions. Thirdly, in previous studies, scholars have often designed questions about health measures from the aspects of “self-evaluation of health” and “mental and emotional”, but these questions have not been uniformly designed, disabling it from global standardization and uniformity, which can easily lead to differences in research conclusions. Consequently, this article analyzes the micro-mechanism of personal endowment and social welfare on the health status of the rural-to-urban elderly migrants based on related theories and construct an analysis framework for the impact of personal endowment and social welfare on the health status of the rural-to-urban elderly migrants. Then, through empirical research, it tests the effects of personal endowment and social welfare on the health status of the migrant elderly in order to improve the health status of the migrant elderly and provide policy suggestions for the government to deepen reform.

## 2. Research Hypothesis and Theoretical Framework

### 2.1. Personal Endowment and Health of Elderly Migrants from Rural Areas

Based on Grossman’s health production function theory, health has the characteristics of both investment products and consumer products. It is mainly affected by family genetic factors, living environment, lifestyle, economic status, education level, emotional stress, and other factors [10]. In the context of this article, the rural-to-urban elderly migrants leave the long-term rural society and follow their children to live in the city. The change of living environment and the transfer of physical space have brought the rural-to-urban elderly migrants into a new living environment, giving rise to new lifestyles and behavior habits. Qin [11] believes that the health resources and human health capital possessed by the migrant population have increased significantly after being resettled into the city. However, due to the inconsistency in the abilities of the individuals of the migrant population to fully own or control such resources, the overall health of the migrant population also presents a level of differentiation. Although the modern urban lifestyle and sufficient medical and health resources meet the needs of the rural migrants for health resources, a small number of the rural-to-urban elderly migrants are unable to control such resources due to insufficient personal financial ability, learning ability, adaptability, etc., which affects their personal or family health status [12]. It can be seen that the personal endowment of the rural-to-urban elderly migrants plays a huge role and influence in the process of obtaining health resources. Therefore, this paper puts forward the research hypothesis:

**Hypothesis** **1** **(H1).** *Personal endowment has a positive impact on the health of elderly migrants from rural areas.*

### 2.2. Social Welfare and Health of Elderly Migrants from Rural Areas

Fletcher [13] found that urbanization provides more opportunities for elderly migrants to obtain urban social capital. However, some rural-to-urban elderly migrants are often “marginalized” after entering the city, and their degree of “citizenization” is not high overall. Psychological gap and loneliness affect the mental health of rural-to-urban elderly migrants. This is mainly reflected in the unequal government welfare policies and differences in the adaptability of the rural-to-urban elderly migrants. In addition, some scholars have found that the willingness of migrants to move with them also affects their personal or family health. Turan [14] pointed out that some farmers are eager to move from the village to the city because they yearn for modern urban lifestyles and medical care resources, showing relatively strong adaptability, and these groups often have good health conditions; Li [15] empirically studied the health status of the elderly in some transitional countries or regions. Due to lack of emotional communication, social welfare deviation, urban employment difficulties, and uncomfortable living environment, some elderly passively moved to cities and lost their original rural areas. At the same time, capital cannot get due to social welfare treatment, resulting in a significant reduction in the health status of this part of the migrant population. Therefore, this paper puts forward the research hypothesis:

**Hypothesis** **2** **(H2).** *Social welfare has a positive impact on the health of elderly migrants from rural areas.*

### 2.3. Theoretical Framework

Based on the above analysis, this article uses the theory of health vulnerability and the theory of risk protection to provide theoretical guidance for explaining the health of rural-to-urban elderly migrants. Health vulnerability essentially refers to the inherent attributes of individuals or families that are highly sensitive to personal health after experiencing system and environmental changes and lack the corresponding adaptability and resistance to cause health damage [16]. Lippke [17] defined the health vulnerability of the rural-to-urban elderly migrants from the perspective of health risks as the level of self-regulation ability after adverse effects. It is not difficult to see that strong sensitivity, low adjustment ability, and poor recovery ability constitute the salient characteristics of fragile health things. In migrating from rural to urban areas, the rural-to-urban elderly migrants have experienced changes in their living environment, family roles, and social group structure. Their physiological characteristics, learning ability, and adaptability determine their relatively strong health and vulnerability characteristics. There will be certain difficulties in resisting the health risks after the social structure changes.

**Figure 1:**
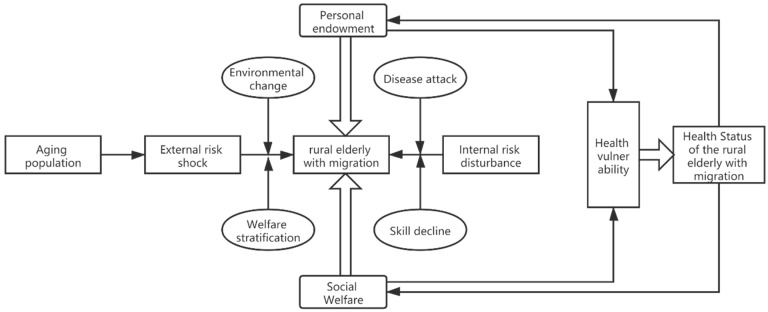
Theoretical analysis framework of the health vulnerability of the rural-to-urban elderly migrants.

Although vulnerability is an ability attribute formed by the long-term accumulation of individuals, Ebi’s research pointed out that through active learning, enhanced adaptability and changes in external factors can reduce their vulnerability, increase risk resistance, and ultimately minimize the health risks caused by vulnerability [18]. The risk protection theory provides an external effect for the rural-to-urban elderly migrants to resist health fragility. Driven by rapid industrialization and urbanization, some rural-to-urban elderly migrants have lost and left the land where they lived for generations and have changed from their traditional farmer status to marginalized citizens. However, the city has corresponding social service policies for the rural-to-urban elderly migrants; due to the lack of natural endowment of the rural-to-urban elderly migrants, the alienation of relatives, and changes in neighboring relationships, these groups face varying degrees of health risks. The continuous improvement of social welfare policies produced certain evasive effects in this process. Under the guidance of the theory mentioned above, this article attempts to construct the following theoretical analysis framework to describe the path of the influence of personal endowment and social welfare on the health of the rural-to-urban elderly migrants (Figure 1).

Based on Figure 1, it is not difficult to find that the external risk impact and internal risk disturbance faced by the rural-to-urban elderly migrants constitute the root of the health vulnerability of the rural-to-urban elderly migrants. The external risks mainly refer to the factors that affect their health caused by environmental changes and the differences in social welfare treatment between urban and rural areas in the transition from traditional farmer status to citizen status for the rural-to-urban elderly migrants. The internal risk is mainly due to the deterioration of their functions due to changes in the living environment and habits of the rural-to-urban elderly migrants after their status changes, as well as reduced health conditions or increased medical expenses due to disease attacks. The health protection system for the rural-to-urban elderly migrants is mainly derived from the combination of personal endowment and social welfare. Personal endowment, as an informal guarantee, presents differentiated characteristics, which are reflected primarily in the ability to deal with human capital, economic capital, and social capital after changes in the living environment. This, in turn, has an important relationship with the willingness of the rural-to-urban elderly migrants to lose their land. The impact of social welfare on the health status of the rural-to-urban elderly migrants is mainly reflected in whether the welfare projects provided by the national macro-welfare policy can effectively reduce the health risks faced by the rural-to-urban elderly migrants or can effectively compensate for the health risks caused by insufficient personal endowments. Specifically, the current state social welfare for the rural-to-urban elderly migrants mainly includes whether to participate in social insurance, whether they can have the same medical facilities, recreational facilities, and job opportunities as urban residents, etc., which can guarantee the availability of medical and health resources for the rural-to-urban elderly migrants. The mechanism of gender and affordability includes the impact of equalization of public services on the mental health of the rural-to-urban elderly migrants.

### 2.4. Research Objective

In summary, this article comprehensively considers the heterogeneity of the personal endowment of the rural-to-urban elderly migrants and the unequal status of social welfare policies in different regions, starting from a multi-measurement dimensional perspective of health, combining the standardized self-rated health scale (SRHMS) and the international used SF-8 scale to comprehensively measure the health status of the rural-to-urban elderly migrants from three perspectives: physical health, mental health, and social adaptation. On this basis, 658 rural migrants from 12 cities (counties) in Jiangsu Province were selected as samples to conduct empirical research to explore the impact of personal endowment and social welfare on the health status of the rural-to-urban elderly migrants the influencing factors of health resource acquisition. As such, this study provides theoretical reference and reference for the government to improve the public welfare policy and improve the health status of the rural-to-urban elderly migrants.

## 3. Methods

### 3.1. Data Sources

Based on theoretical research, this paper analyzes the actual situation of the impact of personal endowment and social welfare on the health of the elderly in rural areas and then designs corresponding questionnaires. In December 2019, the research team distributed 50 questionnaires for pre-survey through field visits in Zhenjiang City. Afterward, the survey data were checked and revised accordingly, and a formal questionnaire was finally formed. From June 2020 to July 2020, the research team selected 12 cities (districts) in Zhenjiang City (Jingkou District, Dantu District, Yangzhong City), Taizhou City (Hailing District, Gaoxin District, Jiangyan District, Xinghua City), Yangzhou (Hanjiang District, Gaoyou City, Yizheng City), and Yancheng (Dongtai City, Dafeng City) in Jiangsu Province as the survey sample areas. Commissioned sophomore students, some graduate students, and teachers from the School of Management of Jiangsu University were trained to visit and fill in the rural-to-urban elderly migrants in the above cities according to the random non-isometric principle. Seven hundred twenty-one questionnaires were issued, and some incomplete information and content were removed. After filling out the conflicting questionnaires, a total of 658 valid questionnaires were obtained, and the effective response rate was 91.26%.

### 3.2. Variable Settings

#### 3.2.1. Health Status

Due to the multiple factors of the formation of the health status of the rural-to-urban elderly migrants and the multidimensional manifestations, the self-evaluated health alone cannot fully measure the health status of the rural-to-urban elderly migrants. Therefore, this article combines the standardized self-rated health measurement scale (SRHMS) and the internationally used SF-8 scale to comprehensively measure the health status of the rural-to-urban elderly migrants from the three perspectives of physical health, mental health, and social adaptation [19,20] and further summarizes the health status of the rural-to-urban elderly migrants into three dimensions: physical health, mental health, and social adaptation.

#### 3.2.2. Personal Endowment

Personal endowment refers to the resources and abilities that an individual has acquired through acquired learning, which mainly includes the three aspects of human capital, economic capital, and social capital obtained. It does not only have an important influence on the health of individuals and family members, but it also has a certain guiding significance for the survival and development and behavioral decision making of family members. For most rural-to-urban elderly migrants who have undergone changes in their living environment, their inherent human capital is gradually alienated, but they will accumulate more or less urban human capital, and occupational differentiation will lead to their human capital, economic capital, and social capital. Corresponding changes will occur, which will affect the health of the rural-to-urban elderly migrants. This article draws on the measurement methods of family endowment by scholars such as Shi and Yue [21,22], from human capital (productive capacity, knowledge stock), social capital (financial support, labor support, emotional support), and economic capital (housing status, material financial accumulation, Monetary Annual Total Income), the three dimensions to construct measurement indicators for the personal endowment of the rural-to-urban elderly migrants.

#### 3.2.3. Social Welfare

The degree of “citizenization” of the rural-to-urban elderly migrants can effectively reflect the degree of social welfare for the rural-to-urban elderly migrants in the process of urbanization. The influence mechanism of social welfare on the health status of the rural-to-urban elderly migrants is mainly embodied in meeting the health and psychological needs of the rural elders through medical security, employment selection, and children’s education. In recent years, although the party and the government have been committed to equalizing public services, the social security system for the rural-to-urban elderly migrants is still not perfect, and the degree of “marginalization” is still relatively serious. This paper chooses two dimensions of social security (whether to participate in social insurance, whether to enjoy local welfare benefits) and social opportunities (medical facilities, recreational facilities, educational resources, job opportunities) to measure the social welfare status of the rural-to-urban elderly migrants. The specific item design is as Table 1, and the questionnaire uses the Likert 5-point scoring method.

## 4. Results

### 4.1. Demographic Structure of the Respondents

The demographic structure of the respondents is shown in Table 2.

### 4.2. Descriptive Statistics and Reliability and Validity Test

The reliability of the scale is mainly tested by Cronbach’s α coefficient. The results show that all α values are greater than 0.7, indicating that the scale’s reliability is good. In addition, the convergent validity and discriminative validity of the measurement terms were tested. The structural relationship between the variables and the measurement terms was initially grasped. In terms of validity test, this paper selects the aggregate validity of the factor loading verification scale and uses whether the square of the variable AVE value is greater than the correlation coefficient between the variables to test the discriminative validity of the scale. The test shows that the factor loadings of all items are greater than 0.6, indicating that the designed questionnaire indicators have good aggregate validity. The square root of the AVE value of each variable in Table 3 is greater than the correlation coefficient between the variables, which shows that the questionnaire indicators designed in this article also have better discriminative validity.

### 4.3. Descriptive Statistics and Correlation Analysis

Table 3 illustrates the statistical results of the mean, standard deviation of each variable, and the correlation between each variable. The rural-to-urban elderly migrants show a general health status, and their personal endowments and social welfare levels are not high. The personal endowment and social welfare of the rural-to-urban elderly migrants are significantly correlated with the three dimensions of health: physical health, mental health, and social health. In order to further verify the intrinsic relationship between personal endowment, social welfare, and the physical health, mental health and social adaptation of the rural-to-urban elderly migrants, the three dimensions of the health status of the rural-to-urban elderly migrants were taken as dependent variables and the personal endowment and social welfare of the rural-to-urban elderly migrants as independent variables. Then, the regression analysis was gradually carried out to explore the influence of personal endowment and social welfare on the health of the rural-to-urban elderly migrants.

### 4.4. Analysis of Regression Results

Taking into account that “the social network between different individuals may lead to differences in the results of actions”, this paper selects the gender, age, marital status, and educational background of the rural-to-urban elderly migrants as the control variables in the regression analysis. Table 4 shows the results of regression model analysis. Model 1, Model 3, and Model 5 are regression models that control the impact of control variables on the health status of rural-to-urban elderly migrants. Model 2, Model 4, and Model 6 are the main effect models of the impact of personal endowment and social welfare on the health status of rural-to-urban elderly migrants.

## 5. Discussion, Suggestion, Limitations

### 5.1. Discussion

With the increasing number and the growing health needs of rural-to-urban elderly migrants, personal endowment and social welfare have become the key factors affecting rural migrant elderly [23]. This study discussed the relationship between personal endowment and social welfare on the health status of the rural-to-urban elderly migrants. According to the analysis results, the age factor in the control variables has a significant negative relationship with the physical health, mental health, and social adaptation of the rural-to-urban elderly migrants, indicating that the older the rural-to-urban elderly migrants, the worse their overall health status. Other variables have insufficient overall explanatory power for the dependent variable regression model of the health status of the rural-to-urban elderly migrants, and they are not statistically significant. The regression results of the main effects model show that by adding two independent variables, personal endowment and social welfare, based on the control variables, the explanatory power of the model has increased significantly. According to Table 4, it can be observed that, in addition to the insignificant impact of personal endowment on the mental health of the rural-to-urban elderly migrants, both personal endowment and social welfare have a significant positive effect on the physical health, mental health, and social adaptation of the rural-to-urban elderly migrants, while Chen (2018) pointed that the mental health of the elderly is affected by endowment factors such as cognitive ability and adaptability [24]. Further comparing the standardized regression coefficients and their significance levels of the two independent variables of the personal endowment and social welfare of the rural-to-urban elderly migrants in Model 2, Model 4, and Model 6, we can summarize as follows.

(1) Personal endowment is the main factor that affects the social adaptation of the rural-to-urban elderly migrants.

The risk of social adaptation for the rural-to-urban elderly migrants is not only reflected in their identity and cultural adaptation risks but also in the emotional or psychological characteristics of individual rural elderly accompanying individuals who have difficulty in social adaptation. Most rural-to-urban elderly migrants still maintain their traditional lifestyles and habits and are caught in the dilemma of interpersonal communication and social adjustment in the new living environment. This is consistent with the prior work of Ziggers (2009), who believes that independent individuals have strong adaptability and can build and manage network relationships helps to improve their success [25]. The application of social relations in communication allows individuals to establish more stable interpersonal relationships [26]. With regard to the efficiency of access to health resources, personal endowments can effectively support the rural-to-urban elderly migrants to grasp and digest external social resources quickly. The environment, behavior, and communication groups of the rural-to-urban elderly migrants living in the city can all change, and the learning can be improved through community guidance or independent learning methods. The enthusiasm and initiative of the villagers can help the rural-to-urban elderly migrants quickly integrate into the new environment and be able to identify and master relevant health knowledge effectively.

(2) Social welfare is the main factor to promote the physical health of rural-to-urban elderly migrants.

The physical health risks faced by the rural-to-urban elderly migrants are mainly reflected in the level of life and employment. The reduction of working hours and changes in living and rest patterns of rural-to-urban elderly migrants after moving to the city, combined with the extravagant consumption (smoking, alcoholism, gambling, increased desire to purchase, etc.) of rural-to-urban elderly migrants, have made rural-to-urban elderly migrants face enormous life-level disease risk. When the rural-to-urban elderly migrants enter a new cultural situation, due to changing the original cultural habits or losing the traditional social foundation, they may encounter a certain degree of cultural conflict or dilemma in the face of changes in the living environment and social roles, resulting in anxiety, worry, and even depression [27]. In addition, the rural-to-urban elderly migrants who leave the land face the transformation of employment patterns from agriculture-related fields to non-agricultural fields, and the lack of human capital and job-selecting ability leads to employment-level disease risks. Enhancing the social welfare for the rural-to-urban elderly migrants can enhance the participation rights of the rural-to-urban elderly migrants, alleviate employment difficulties, and protect the health of residents. Model 2 further verifies that social welfare has a relatively important impact on the physical health of the rural-to-urban elderly migrants and can provide basic guarantees for the employment, education, and health of the rural-to-urban elderly migrants.

(3) Social welfare is also a major factor in promoting the mental health of rural-to-urban elderly migrants.

Changes in the living environment are one of the main factors that affect mental health. The rural-to-urban elderly migrants not only face changes in their production and lifestyles but also face the process of changing their identities from rural residents to urban residents. The rural-to-urban elderly migrants are often considered “marginalized” groups outside of urban residents. Insufficient participation in society, difficulty in choosing jobs, and differences in economic status can easily lead to psychological isolation, hostility, terror, or mental illnesses such as paranoia. Therefore, in the study of the relationship between urbanization and health in China, the degree of urbanization has a negative impact on residents’ health system [28], and further improving the social security policies for the rural-to-urban elderly migrants and ensuring the equalization of public services for the rural-to-urban elderly migrants and urban residents can help improve the psychological identity of the rural-to-urban elderly migrants and the positive acceptance of urban residents.

### 5.2. Suggestion

(1) Cultivating the learning ability of the rural-to-urban elderly migrants. The learning ability can effectively improve the personal endowment of the rural-to-urban elderly migrants and help the rural-to-urban elderly migrants to grasp and digest external social resources quickly. The living environment, behaviors, and people with whom the rural-to-urban elderly migrants enter the city will change. Improving the enthusiasm and initiative of learning through community guidance or adopting independent learning methods will help the rural-to-urban elderly migrants quickly integrate into the new environment and can effectively identify and master relevant health knowledge and obtain effective health resources.

(2) Promoting the equalization of public services. Generally speaking, it has become an indisputable fact that the degree of “citizenization” of the rural-to-urban elderly migrants after changing their status as “farmers” is still not high. The marginalization of the rural-to-urban elderly migrants will have a huge impact on their health, especially social adaptation. Further improving the welfare policies for the rural-to-urban elderly migrants and ensuring equal employment, medical care, education, and other public services for the rural-to-urban elderly migrants and urban residents will help improve the psychological identity of the rural-to-urban elderly migrants and the positive acceptance of urban residents.

(3) Establishing a community-based health promotion system for the rural-to-urban elderly migrants. The health department can take the initiative to publicize basic public health knowledge, increase health education and training guidance for the rural-to-urban elderly migrants, and improve their disease prevention and response capabilities. The community can take active publicity and education methods to guide the rural-to-urban elderly migrants to get used to the urbanized lifestyle and integrate into the urban life circle as soon as possible. Other relevant social organizations can also build a community-based mutual aid communication platform on encouraging rural-to-urban elderly migrants to make new social capital, form a mode of interpersonal communication, enhance the social adaptability of the rural-to-urban elderly migrants, and improve their physical and mental health.

### 5.3. Limitations

Given the large data set, a sample of rural-to-urban elderly migrants from the Jiangsu province is included in this research. It would be useful to replicate this study using Jiangsu province measurement methods in China’s other provinces to boost findings’ generalizability.

## 6. Conclusions

This paper constructs a theoretical analysis framework on the basis of theoretically discussing the impact of personal endowment and social welfare on the health status of rural-to-urban elderly migrants. It uses 658 data of rural-to-urban elderly migrants in Jiangsu Province to conduct empirical research and examines personal endowment and society through the main effect regression analysis method. The impact and difference of welfare on the health status of rural-to-urban elderly migrants. The research conclusions mainly include:

(1) Insufficient personal endowment hinders the social adaptability of the rural-to-urban elderly migrants. Social adaptation helps to enhance the cognitive paradigm of the rural-to-urban elderly migrants and enhance the understanding and recognition of the rural-to-urban elderly migrants in the organization. However, the foundation of personal endowment lies in learning. According to statistics, the number of rural-to-urban elderly migrants over 75 years old accounted for 38.5% of the total sample. The interview revealed that these groups have lived in rural areas for generations and have a strong “native” affection, making them more willing to maintain their inherent rural lifestyle habits. In the sample, as many as 81% of the rural migrants with junior high school and below education level, this also shows to a certain extent that this group has insufficient learning ability. In addition, because health often has the characteristics of delay and uncertainty, strong social adaptability usually requires rural-to-urban elderly migrants to consume more time and energy. The tacit attributes of the personal endowment will also to a certain extent affect the social adaptability of the rural-to-urban elderly migrants.

(2) The imperfect social welfare policy restricts the physical and mental health of characteristics attributed to rural-to-urban elderly migrants. Medical security, public facilities, employment resources, etc., constitute the main part of the social welfare for the rural-to-urban elderly migrants. On the whole, the rural-to-urban elderly migrants in the sample have average evaluations on the medical security, employment, and public facilities they have obtained after living in the city. Considering the changes in lifestyle and economic sources of the rural-to-urban elderly migrants, the lack of medical insurance will restrict the physical health of the rural-to-urban elderly migrants. The difficulty in choosing a job due to insufficient human capital, coupled with the difference in the supply of public facilities between urban residents and rural migrants, will also impact the mental health of the rural-to-urban elderly migrants.

## Figures and Tables

**Table 1:** Reliability and validity analysis of variables.

Variable	Item	Factor Loading	Cronbach’s α Coefficient	Cumulative Explanatory Variance (%)
Physical health	Feel healthy	0.759	0.930	83.009
	The physical condition will not affect general physical activity	0.901		
	Health status will not affect daily work	0.880		
	No physical pain in the last four weeks	0.780		
Mental health	Always feel energetic	0.970	0.970	94.295
	Always feel happy	0.926		
	Always feel that life is fulfilling	0.932		
Social adaptation	Did not affect the general schedule activities of family members or friends due to their own health conditions	0.684	0.875	80.871
	Did not affect daily work and activities due to emotional distress	0.878		
	Daily work or activities will not be disturbed by the emotions of others	0.864		
	Able to get along well with neighbors and friends	0.771		
Personal endowment	Work and exercise every day	0.646	0.731	67.882
	Keep learning every day	0.941		
	It is easy to get help from friends when you need to borrow money	0.874		
	When production or labor encounters difficulties, it is easy to get help from friends	0.710		
	When you encounter major decisions in production or life, you can easily get suggestions from friends	0.744		
	Very satisfied with the current living environment and conditions	0.809		
	Very satisfied with personal and family material wealth	0.890		
	Personal annual monetary income meets life needs and has a balance	0.829		
Social welfare	Able to participate in local social insurance	0.836	0.919	76.328
	Able to enjoy the same benefits as local residents	0.807		
	Convenient medical treatment in the community where you live	0.852		
	The community is equipped with relevant entertainment and fitness facilities	0.726		
	Sufficient educational resources in the community	0.802		
	Finding a job locally is not difficult	0.629		

**Table 2:** Demographic structure of the respondents.

Variables		Number (Person)	Ratio (%)	Variables		Number (Person)	Ratio (%)
Gender	Male	337	51.22	Monthly household income	≤1000 yuan	217	32.98
	Female	321	48.78		1001–3000 yuan	173	26.29
Age	60–65 years	239	36.32		3001–5000 yuan	192	29.18
	66–70 years	168	25.53		>5000 yuan	76	11.55
	71–75 years	78	11.85	Registered residence	Town	459	69.76
	76–80 years	92	13.98		Countryside	199	30.24
	>80 years	81	12.31	Pre-retirement occupation	Farmer	393	59.73
Education	Primary school or below	214	32.52		Construction worker	137	20.82
	Middle school	209	31.76		Manufacturing	56	8.51
	High school	136	20.67		Service industry	32	4.86
	Junior college	79	12.01		Other	40	6.08
	University and above	20	3.04	Marital status	Unmarried, divorced, or widowed	169	25.68
					Married	489	74.32

**Table 3:** Descriptive statistics and correlation analysis.

Variable	Mean	Standard Deviation	1	2	3	4	5
1 Physical health	3.123	0.905	**0.893**				
2. Mental health	3.426	0.939	0.823 ***	**0.889**			
3. Social adaptation	3.532	0.849	0.721 ***	0.676 ***	**0.762**		
4. Personal endowment	2.946	0.658	0.715 ***	0.563 ***	0.489 ***	**0.771**	
5. Social welfare	2.864	0.902	0.715 ***	0.681 ***	0.509 ***	0.286 *	**0.789**

Note: *** *p* < 0.01; * *p* < 0.1; the bolded diagonal value is the square root of the AVE value.

**Table 4:** Analysis of regression results.

Variable	Physical Health		Mental Health		Social Adaptation	
	Model 1	Model 2	Model 3	Model 4	Model 5	Model 6
Gender	0.72	0.109 **	0.142 *	0.164 **	0.002	0.029
Age	−0.574 ***	−0.259 ***	−0.535 ***	−0.273 **	−0.363 **	−0.222 **
Marital status	−0.107	−0.104	−0.271 *	−0.244 *	0.224 *	0.198 *
Education level	0.027	−0.025	−0.043	−0.081 *	−0.052	−0.081 *
Personal endowment		0.186 **		0.061		0.191 ***
Social welfare		0.444 ***		0.424 ***		0.135 *
R^2^	0.620	0.721	0.445	0.512	0.295	0.321
Adjusted R^2^	0.617	0.719	0.442	0.508	0.291	0.315
F	266.097 ***	280.747 ***	130.935 ***	113.945 ***	68.343 ***	51.250 ***

Note: *** *p* < 0.01; ** *p* < 0.05; * *p* < 0.1.

## Data Availability

The data for this research are held by the authors and will be made available upon reasonable request.
